# Supplementary material for: Experimental demonstration of a skyrmion-enhanced strain-mediated physical reservoir computing system
Source: Nat Commun. 2023 Jun 10;14:3434. doi: 10.1038/s41467-023-39207-9 (PMC10257712; doi:10.1038/s41467-023-39207-9)
Supplement: Supplementary file 1 — Supplementary Information [file 41467_2023_39207_MOESM1_ESM.pdf]

Supplementary Information:

S1: Confirmation of the interfaces' quality

A cross-sectional transmission electron micrograph (TEM) measurement is conducted to confirm the quality of the interfaces, as shown in Fig. S1a, with the sample structure of Si/Ta (2 nm)/Pt (3 nm)/[Co (1.95 nm)/Gd (1.2 nm)/Pt (3 nm)]$_7$. The multilayers are polycrystalline with sharp interfaces as can be seen in the high angle annular dark-field scanning transmission electron microscopy (HAADF-STEM) image together with the energy dispersive X-ray (EDX) mapping shown in Figs. S1b and S1c. The periodical Pt/Co/Gd multilayer structure is further confirmed in Fig. S1c. The Pt/Co interface is sharper than the Gd/Pt interface, which is mainly because the Gd layer is very thin (1.2 nm) and also because Gd atom easily diffuse. These distinct interfaces ensure the formation of the skyrmions.

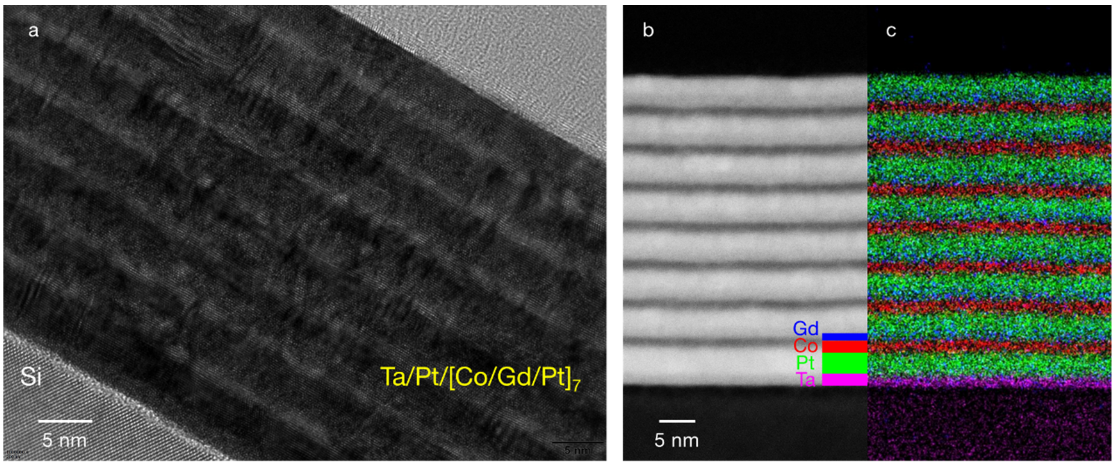

Fig. S1 Characterization of the sample's structure. a, The cross-sectional TEM image of the multilayer. **b,** The cross-sectional HAADF-STEM image of the multilayer. **c,** The corresponding EDX mapping for element distributions of Pt (green), Co (red), Gd (blue) and Ta (magenta). The scale bar is 5 nm for **a, b** and **c**.

S2: Magnetic properties and micromagnetic structures observation of the sample

The sample structure and its hysteresis loop under out-of-plane magnetic fields, measured using polar MOKE at room temperature, are shown in Fig. S2a.

L-TEM measurements are conducted to observe the micromagnetic structures and confirm the skyrmion type in this multilayer structure. A sample deposited on a 50-nm-thick Si_3N_4 membrane is used for L-TEM measurements, with a tilting angle of 10°, as shown in Fig. S2b. Upon increasing the magnetic field, the evolution of the magnetic textures is visible, from labyrinth domains (I), then shrinking to magnetic skyrmions (II), and finally to a fully saturated single-domain state (III). Néel-type skyrmions in the Pt/Co/Gd multilayers are confirmed from the L-TEM images.

The alloy/multilayers of rare earth and transition metal are typically ferrimagnetic systems [1]. Here, the temperature-dependence of the magnetization in the Pt/Co/Gd multilayers is measured by superconducting interference device vibrating sample magnetometry (SQUID-VSM), as shown in Fig. S2c. M_s decreased with temperature, indicating compensation between the Co and Gd layers due to the antiferromagnetic coupling. We conclude that ferrimagnetic skyrmions exist in the Pt/Co/Gd multilayers.

The out-of-plane hysteresis loops at different temperatures are shown in the inset; the loops maintain the characteristic sheared shape down to 25 K. Thus, we can expect ferrimagnetic skyrmions over the full temperature range from 25 K to room temperature.

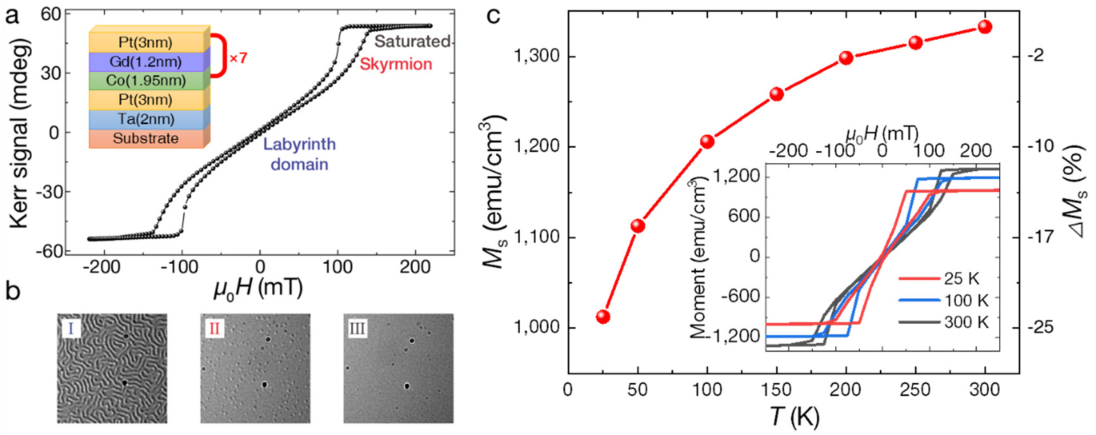

Fig. S2 Sample information and skyrmion observations. a, Out-of-plane magnetic hysteresis measured at room temperature – the inset shows the sample configuration. **b,** L-TEM images demonstrating magnetic structures in the multiferroic heterostructure. The image size is 5×5 μm^2. **c,** Saturation magnetization-temperature relationship of the ferrimagnetic skyrmion structure. The inset shows the magnetic hysteresis at different temperatures measured by SQUID-VSM.

S3: Relations between the *E*-field controlled skyrmion density change and magnetization change

In order to count the skyrmion area from the MFM images in the Fig. 2a insets I and II, we binarize the MFM images as shown in Figs. S3a and S3b, and the corresponding $\Delta M_{skyrmion}/M_s$ are 1.3% and 13% summarized from the black area, respectively. $\Delta M(E)$ for *E*-field between 0 and 10 kV/cm is 11.7%. This result is in a good agreement with the results shown in Fig. 2b (M/M_s extracted from the hysteresis loops measured by MOKE in Fig. 2a), which is $\frac{92\%-66\%}{2} = 13\%$. The skyrmions density increase gives rise to the magnetization decrease in the MOKE measurement. The $\Delta M(E)$ at $\mu_0 H_a = 90$ mT shown in Fig. 2b represents the skyrmion generation with increasing the *E*-field.

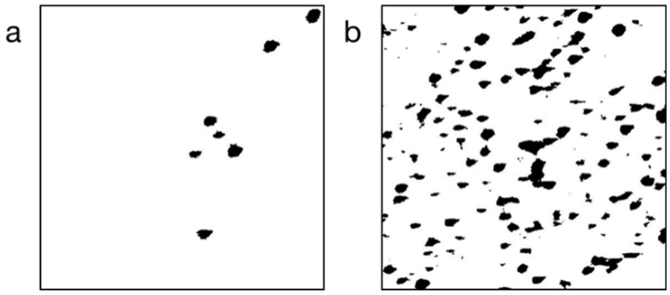

Fig. S3 Binarized results of the inset MFM images in Fig. 2a. a and **b** are corresponding to the stages I and II, respectively. The image size is 5×5 μm^2.

S4: Strain-induced magnetic anisotropy change

MOKE with rotating magnetic fields (Rot-MOKE) is conducted to evaluate the effective magnetic anisotropy fields quantitatively with the applied *E*-fields. For Rot-MOKE experiments, we start rotating

from the polar-MOKE configuration as illustrated in Fig. S4a. Figure S4b shows the experimental (data points) and fitting (curves) results of the torque $l(\theta)$ as a function of the magnetization equilibrium angle θ under different applied E-fields. The effective magnetic anisotropy fields ($H_{k,eff}$) are −267, −504 and −731 Oe for 0, −10 and −20 kV/cm, respectively. A negative $H_{k,eff}$ is obtained, due to the in-plane magnetic anisotropy in Pt/Co/Gd multilayers. The in-plane magnetic anisotropy presumably originates from the strong dipolar interaction due to the multi-repetitions, since the trilayer of Pt/Co/Gd shows clear out-of-plane easy axis loop in Fig. S4c. The change of magnetic anisotropy fields $\Delta H_k(E) = H_{k,eff}(E) - H_{k,eff}(E_0)$ is plotted as a function of the sweeping E-fields, shown in Fig. S4d. A butterfly shape (typical strain-E relationship of PMN-PT substrates) with the same switching fields (±1.67 kV/cm) is shown in Fig. 2b, meaning that the anisotropy change originates from the strain generated by E-fields.

Here, the effective magnetic anisotropy is $K_{eff} = K_u - \frac{\mu_0 M_s^2}{2} = \frac{\mu_0 M_s H_{k,eff}}{2}$, where K_u is the uniaxial magnetic anisotropy, μ_0 is vacuum permeability and M_s is the saturation magnetization. Utilizing the strain-mediated magnetoelectric coupling in the piezoelectric/ferrimagnetic heterostructure, the simplified strain-induced anisotropy is written as $K_e = -\frac{3}{2}\lambda\sigma$, $\sigma = \varepsilon E_f/(1-\nu^2)$, where λ is the magnetostriction coefficient, ε is the strain, E_f is the Young's modulus, and ν is the Poisson ratio [2]. For Pt/Co/Gd multilayers, we take E_f = 168, 209, and 55 GPa for Pt, Co and Gd to obtain the effective Young's modulus, ν = 0.3 for metals and $\lambda = -1.7 \times 10^{-4}$ for $GdCo_2$ alloy [3,4]. For the PMN-PT (100) single crystal, ε = 0.2% at ±20 kV/cm is taken [5]. The strain-induced anisotropy change in the Pt/Co/Gd multilayer can be estimated: $K_e = 8.5 \times 10^4$ J/m^3. The equivalent anisotropy field change is $\Delta H_e = \frac{2K_e}{\mu_0 M_s}$ =1416.7 Oe, where the measured $M_s = 1.2 \times 10^6$ A/m. The measured $\Delta H_{k,eff}$ = 464 Oe (between 0 and −20 kV/cm) is lower than the estimated value, which is presumably due to the transferred strain loss in the multilayers compared to the PMN-PT substrate. Additionally, for the positive piezoelectric coefficients d_{33} in PMN-PT, a tensile strain in out-of-plane direction is generated when the E-field is higher than the ferroelectric switching field, resulting in an in-plane compressive strain. Considering the strain transferred from PMN-PT to the multilayers on top, together with the negative λ in CoGd, a decrease of the out-of-plane magnetic anisotropy can be concluded. Namely, the in-plane magnetic anisotropy increases with E-fields, which is consistent with the measured results, as the negative ΔH_k shown in Fig. S4d.

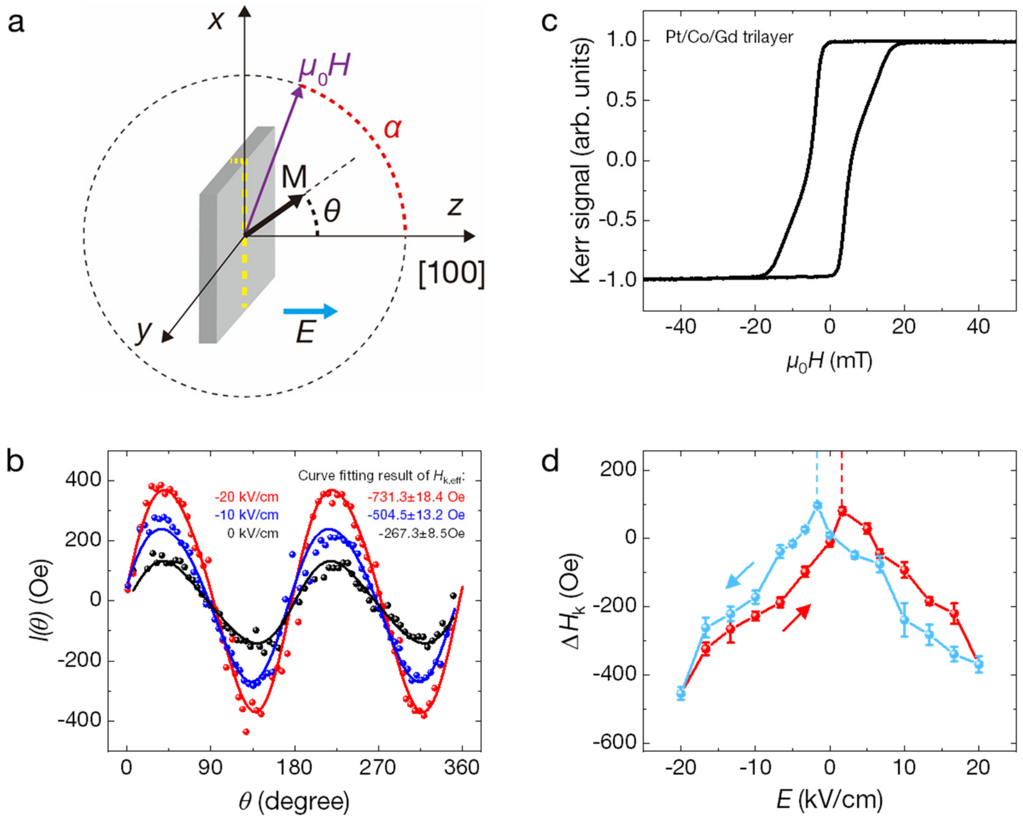

Fig. S4 Evaluation of magnetic anisotropy changes with *E*-fields. a, Sketch of Rot-MOKE measurements. **b,** The torque $l(\theta)$ versus θ with different E-fields, the solid curves are fitting results. **c,** Out-of-plane hysteresis loop for the Pt/Co/Gd trilayer sample, where the thickness of the Co layer is 1.95 nm. **d,** Fitting results of the magnetic anisotropy field change ΔH_k as a function of the E-fields. The arrows indicate the E-field sweeping direction.

S5: Evaluation of DMI in Pt/Co/Gd trilayer

Measurements for DMI in Pt/Co/Gd trilayer are conducted by the spin wave dispersion-based Brillouin light scattering (BLS) method [6]. The sample structure is Si/Pt (5 nm)/Co (2.2 nm)/Gd (2 nm)/Pt (2 nm), where the cobalt thickness is thicker than that of the multilayer stack with ferrimagnetic skyrmions in this work in order to achieve in-plane magnetic anisotropy. The BLS spectrum is shown in Fig. S5a, the peaks are well fitted by the Lorentzian curves. The Δf (frequency shift)- k (wave vector) relationship shown in Fig. S5b exhibits a good linearity. The saturation magnetization M_s is measured as 2.57×10^5 A/m by vibrating sample magnetometer (VSM). According to $\Delta f = \frac{2\gamma}{\pi M_s} D k$ [7], where γ is the gyromagnetic ratio, taking $\frac{\gamma}{2\pi}$ = 30.35 GHz/T [8], the calculated DMI constant D is −0.29 mJ/m^2 for the Pt/Co/Gd trilayer. This result is comparable with other Pt/Co based skyrmion materials, such as Pt/Co/W multilayers [9].

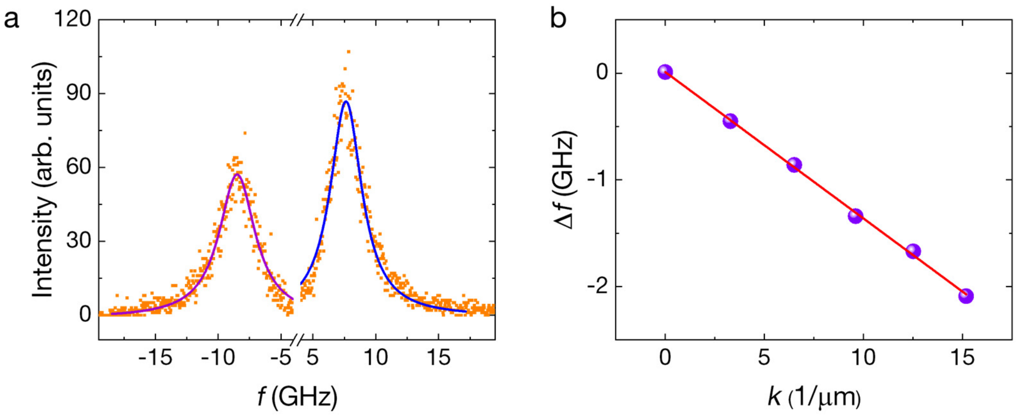

Fig. S5 Evaluation of DMI in Si/Pt(5 nm)/Co(2.2 nm)/Gd(2 nm)/Pt(2 nm). a, Dots: BLS spectrum at the incident angle of 16 degrees. Lines: Lorentzian peak fit for the Stokes (left) and anti-Stokes (right) components of the BLS spectrum. **b,** Frequency shifts versus wave vectors. Red line: Linear fitting of the data.

S6: Constructed model for Mackey-Glass time series prediction task

When studying a more complex task like the MG time series prediction task, the noise of the measurement system is on a sub μV level, which submerges the small signal information since the peak-to-peak signal is a few μV. In this case, we take advantage of the Neural ODE to build an ideal (noiseless) model for our nonlinear system to perform the complex MG task, in order to give an instruction for the experiments

Based on experimental data of the waveform recognition task, we develop a model to precisely describe the nonlinear relation between the input voltage V(t) and output voltage V_{xy}. The model is built by using the latest deep learning method called Neural Ordinary Differential Equations (ODEs) [10]. The method is based on a small amount of sample data to train an ODE $\dot{V}_{xy}(\mathrm{t}) = f(V_{xy}(\mathrm{t}), V(\mathrm{t});\ \theta)$ in which the function f is described by a neural network with parameter θ (see below for model details). The ODE chosen as the modelling frame is based on the fact that the present-time output voltage is dependent on both the present-time input into the system and the previous output voltage from the system. The testing results of Neural ODE output and the experimental output show good agreement with each other, as shown in Fig. S6a. Furthermore, Fig. S6b shows the comparison of the recognition rate between the experimental results and the Neural ODE model results, which also exhibits high consistency. The trained model is then evaluated for the MG prediction task.

The goal of the task is to predict the next steps in the time series. Here, the previously trained model of skyrmion-enhanced strain-mediated reservoir is applied for the task. The detailed procedure and parameters for solving the prediction task can be found in Ref. [10]. The prepared MG series contains 10,000 data points. The first 5,000+i data points are used for the training, and the rest 5,000−i points are as testing set. Figure S6c and S6d show the selected predicting results of MG time series for the next value (i = 1), and the value happening 25 steps later (i = 25), respectively. Each horizontal prediction step i corresponds to a different prediction task. In general, it is more difficult to predict for larger steps due to the chaotic nature of the MG time series. In the situation of i = 1, the predicting results from the model match the ground truth perfectly, while for i = 25, a small prediction error happens. Figure S6e presents the

accuracy of the MG series prediction, expressed in terms of normalized root mean square error (NRMSE) in a logarithmic scale as a function of *i*. This demonstration of a demanding benchmark task indicates the great potential of the skyrmion-enhanced strain-mediated reservoir system for more complex tasks.

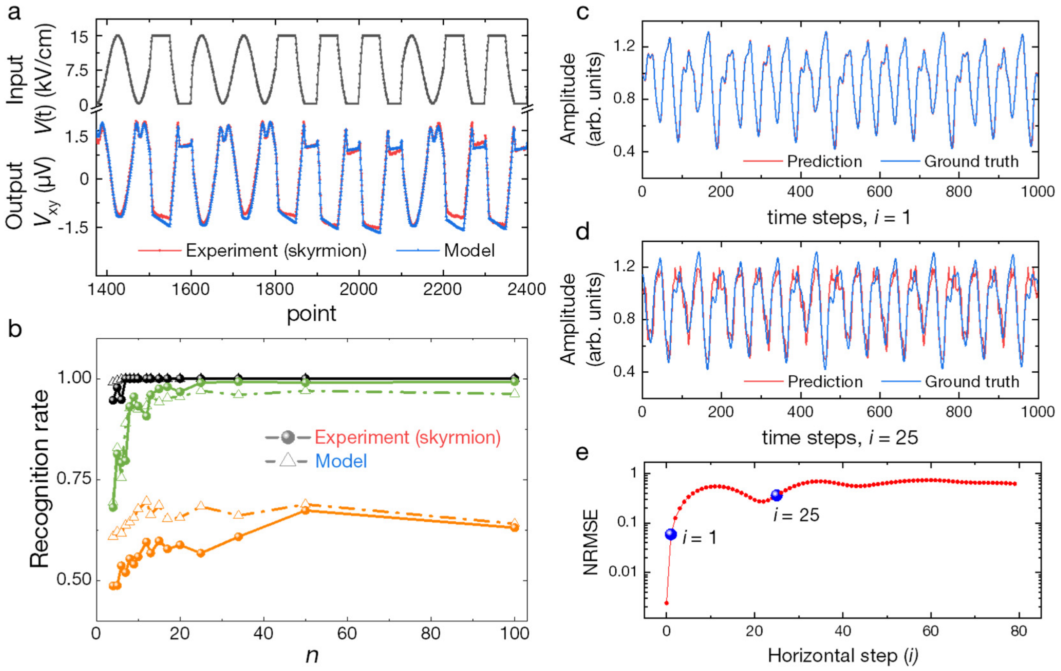

Fig. S6 Mackey-Glass time series prediction task via the constructed model of skyrmion-enhanced strain-mediated RC system. a, Input waveform sequences and the corresponding output signals of the strain-mediated reservoir. The blue and red output signals correspond to the Neural ODE model and experimental results at skyrmion state. **b,** Recognition rates as a function of the number of data points sampled per period for experiment (skyrmion state) and Neural ODE model of the strain-mediated RC. They all match closely for the current (black), last (green), and second-to-last (orange) waveforms. **c** and **d** are the selected predicting (red) results for horizontal step $i = 1$ (for short-term prediction) and 25 (for long-term prediction), predicted by the skyrmion-enhanced strain-mediated RC system modelled by Neural ODE, in comparison with the ground truth (blue) of MG time series. **e,** NRMSE as a function of horizontal prediction step *i*, shown in log scale, for the testing set by using the Neural ODE model.

The prediction performance of the experiment behaves similarly with the constructed model by Neural ODE, as compared in Fig. S7.

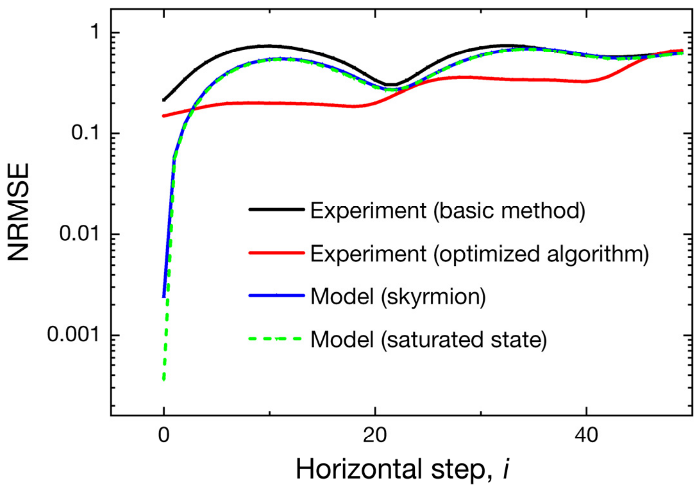

Fig. S7 MG time series prediction task performance of the strain-mediated spintronic RC system and the constructed model. The black and red curves are calculated from experimental data (the same with Fig. 4b). And the blue curve and the green dashed curve are the result of constructed models by Neural ODE for skyrmion and saturated states, respectively.

The following are the methods used in the construction of Neural ODE model.

We use the experimental data from the waveform recognition task to model the skyrmion-enhanced strain-mediated RC system. A single-trajectory training set y_{true} (consisting of k continuous data points from the output trajectory V_{xy}(t) with corresponding k data points from V(t) as input) is built to train the Neural ODE [6]. The mean squared error (MSE) between the experimental data and the corresponding trajectories predicted by the Neural ODE y_{pred} over all time steps is set as the "loss function". The training process aims to achieve the minimization of the loss, where the gradients of the loss with respect to the parameters θ are computed through a technique called adjoint sensitivity method [11]. Then the parameters can be updated by using gradient descent optimization algorithms until the MSE approaches zero. In this work, we train a Neural ODE in the form of $\dot{V}_{\mathrm{xy}}(\mathrm{t}) = f(V_{\mathrm{xy}}(\mathrm{t}), V(\mathrm{t});\ \theta)$, where f function of the Neural ODEs is a three-layer feedforward neural network with each hidden layer featuring 50 units. The activation function is the tanh function except for the output layer.

The number of training data points k = 10,000 and validation data points of 5,000 are used for the skyrmion-enhanced strain-mediated RC system. Once the training is finished, the prediction is made by specifying an initial value of V_{xy}(t) and applying the time-varying inputs V(t) into the trained Neural ODE. Here, the test set of the total 40,000 points for the whole waveform recognition task is used to evaluate the prediction performance of the trained Neural ODE.

S7: Nonvolatile resistivity controlled by strain

As shown in Fig. S8a, the looplike response of the AHE signal ΔV_{xy} appears after applying a negative polarized electric-field, indicating that the 109° ferroelastic switching is the dominating factor in our system. And this non-volatile behaviour still remains with sweeping the E-field in positive range (Fig. S8b). The nonvolatility of the AHE signal makes it possible to be an adequate physical reservoir with memory effect.

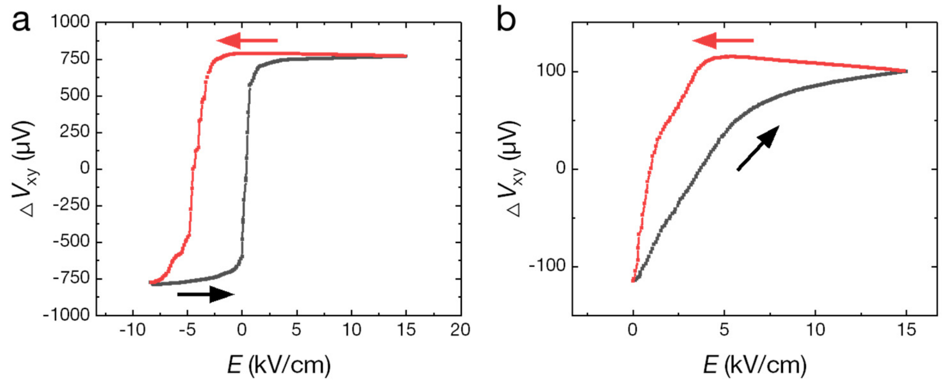

Fig. S8 Nonvolatility of the AHE signal ΔV_{xy} after applying the negative *E*-field. a, *E*-field between -8.3 to 15 kV/cm. **b,** *E*-field between 0 to 15 kV/cm after the first cycle shown in **a**.

S8: Energy dissipation estimation

The operation energy dissipated in the system is $E_s = \frac{1}{2}CV^2$, where C is the crystal capacitance of the PMN-PT layer and the V stands for voltage amplitude [12]. The capacitance of the PMN-PT layer is around 1.7 fF for a 100 nm diameter nanomagnet (for 10 nm-scale skyrmions [13], it is large enough to ensure a sufficient quantity of skyrmions to possess nonlinearity, complexity and short-term memory properties) and a large relative dielectric constant of 2900 for PMN-PT at room temperature [14]. A voltage pulse of ±50 mV (with PMN-PT thickness of 100 nm [15] corresponding to ±5 kV/cm) is required for waveform classification tasks. For a total energy dissipation of $2CV^2 = 850\ \mathrm{aJ}$ per waveform in 1 ns (1 GHz of ferromagnetic materials natural frequency), the power dissipation will be 850 nW, which is considerably low.

The ferroelectric hysteresis loss is determined by integrating the area of the *P-E* hysteresis loop using Greene's theorem. In our experimental process, the unipolar sweeping of the *E*-fields was performed. For (001)-oriented PMN-30%PT, the unipolar *P-E* response has been studied [16]. Since barely hysteretic *P-E* responses driven by the unipolar electric field, the total hysteresis loss is estimated to be less than 2000 J/m^3 per cycle. Considering the nanodevice we propose above, the estimated energy dissipation from ferroelectric hysteresis loss will be less than 6.28 aJ.

The readout energy cost in the AHE measurements with a DC current of I_{dc} = 0.5 mA, and the output V_{xy} of less than 50 mV, is about 25 μW coming into the Joule heating on the Hall bar device. When decreasing the device size down to a 100 nm diameter nanomagnet, the cross-area of the Hall bar will be (100 nm)^2/10 μmð 150 μm = 1/150000 of the original size. The energy dissipation will reduce to 1/150000 of 25 μW, i.e., 166.7 pW. Considering the 1 GHz waveform signal, the power dissipation is much less than the previous estimation of 850 nW.

In sum, the total energy dissipation for the waveform classification task is less than 1 fJ per waveform.

Supplementary References

1 González, J. A., Andrés, J. P., & López Antón, R. Applied Trends in Magnetic Rare Earth/Transition Metal Alloys and Multilayers. *Sensors* **21**, 5615 (2021).

2 Lei, N., Park, S., Lecoeur, P., Ravelosona, D. & Chappert, C. Magnetization reversal assisted by the inverse piezoelectric effect in Co-Fe-B/ferroelectric multilayers. *Phys. Rev. B* **84,** 012404 (2011).

3 https://periodictable.com/Properties/A/YoungModulus.html.

4 Lee, E. W. & Pourarian, F. Magnetoelastic properties of (Rare-Earth)–Co_2 compounds. II. The anisotropic magnetostriction. *Phys. Stat. Sol. (a)* **34,** 383 (1976).

5 Sabolsky, E.M. et al. Piezoelectric properties of <001> textured $Pb(Mg_{1/3}Nb_{2/3})O_3$–$PbTiO_3$ ceramics. *Appl. Phys. Lett.* **78,** 2551 (2001).

6 Nembach, H. T., Shaw, J. M., Weiler, M., Jué, E. & Silva, T. J. Linear relation between Heisenberg exchange and interfacial Dzyaloshinskii–Moriya interaction in metal films. *Nat. Phys.* **11**, 825 (2015).

7 Di, K. et al. Asymmetric spin-wave dispersion due to Dzyaloshinskii-Moriya interaction in an ultrathin Pt/CoFeB film. *Appl. Phys. Lett.* **106**, 052403 (2015).

8 Belmeguenai, M. et al. Influence of the capping layer material on the interfacial Dzyaloshinskii–Moriya interaction in Pt/Co/capping layer structures probed by Brillouin light scattering. *J. Phys. D Appl. Phys.* **52**, 125002 (2019).

9 Lin, T. et al. Observation of room-temperature magnetic skyrmions in Pt/Co/W structures with a large spin-orbit coupling. *Phys. Rev. B* **98**, 174425 (2018).

10 Chen, X., Araujo, F.A., Riou, M. et al. Forecasting the outcome of spintronic experiments with Neural Ordinary Differential Equations. *Nat. Commun.* **13**, 1016 (2022).

11 Chen, T. Q., Rubanova, Y., Bettencourt, J. & Duvenaud, D. K. Neural ordinary differential equations. *Adv. Neural Inf. Process. Syst.* **31**, 6571 (2018).

12 D′Souza, N., Atulasimha, J. and Bandyopadhyay, S. Four-state nanomagnetic logic using multiferroics. *J. Phys. D: Appl. Phys.* **44**, 265001 (2011).

13 Meyer, S., Perini, M., von Malottki, S. et al. Isolated zero field sub-10 nm skyrmions in ultrathin Co films. *Nat. Commun.* **10**, 3823 (2019).

14 Sabolsky, E.M. et al. Piezoelectric properties of <001> textured $Pb(Mg_{1/3}Nb_{2/3})O_3$–$PbTiO_3$ ceramics. *Appl. Phys. Lett.* **78**, 2551 (2001).

15 Baek, S. H. et al, Giant Piezoelectricity on Si for Hyperactive MEMS. *Science* **334**, 958 (2011).

16 Viehland, D. Effect of uniaxial stress upon the electromechanical properties of various piezoelectric ceramics and single crystals. *J. Am. Ceram. Soc.* **89**, 775 (2006).
